# Delivery channels and socioeconomic inequalities in coverage of reproductive, maternal, newborn, and child health interventions: analysis of 36 cross-sectional surveys in low-income and middle-income countries

**DOI:** 10.1016/S2214-109X(21)00204-7

**Published:** 2021-05-26

**Authors:** Daniel G P Leventhal, Inácio Crochemore-Silva, Luis P Vidaletti, Nancy Armenta-Paulino, Aluísio J D Barros, Cesar G Victora

**Affiliations:** a International Center for Equity in Health, Federal University of Pelotas, Pelotas, RS, Brazil

## Abstract

**Background:**

Global reports have described inequalities in coverage of reproductive, maternal, newborn, and child health (RMNCH) interventions, but little is known about how socioeconomic inequality in intervention coverage varies across multiple low-income and middle-income countries (LMICs). We aimed to assess the association between wealth-related inequalities in coverage of RMNCH interventions.

**Methods:**

In this cross-sectional study, we identified publicly available Demographic Health Surveys and Multiple Indicator Cluster Surveys from LMICs containing information on household characteristics, reproductive health, women's and children's health, nutrition, and mortality. We identified the most recent survey from the period 2010–19 for 36 countries that contained data for our preselected set of 18 RMNCH interventions. 21 countries also had information on two common malaria interventions. We classified interventions into four groups according to their predominant delivery channels: health facility based, community based, environmental, and culturally driven (including breastfeeding practices). Within each country, we derived wealth quintiles from information on household asset indices. We studied two summary measures of within-country wealth-related inequality: absolute inequalities (akin to coverage differences among children from wealthy and poor households) using the slope index of inequality (SII), and relative inequalities (akin to the ratio of coverage levels for wealthy and poor children) using the concentration index (CIX). Pro-poor inequalities are present when intervention coverage decreased with increasing household wealth, and pro-rich inequalities are present when intervention coverage increased as household wealth increased.

**Findings:**

Across the 36 LMICs included in our analyses, coverage of most interventions had pro-rich patterns in most countries, except for two breastfeeding indicators that mostly had higher coverage among poor women, children and households than wealthy women, children, and households. Environmental interventions were the most unequal, particularly use of clean fuels, which had median levels of SII of 48·8 (8·6–85·7) and CIX of 67·0 (45·0–85·8). Interventions primarily delivered in health facilities—namely institutional childbirth (median SII 46·7 [23·1–63·3] and CIX 11·4 [4·5–23·4]) and antenatal care (median SII 26·7 [17·0–47·2] and CIX 10·0 [4·2–17·1])—also usually had pro-rich patterns. By comparison, primarily community-based interventions, including those against malaria, were more equitably distributed—eg, oral rehydration therapy (median SII 9·4 [2·9–19·0] and CIX 3·4 [1·3–25·0]) and polio immunisation (SII 12·1 [2·3–25·0] and CIX 3·1 [0·5–7·1]). Differences across the four types of delivery channels in terms of both inequality indices were significant (SII p=0·0052; CIX p=0·0048).

**Interpretation:**

Interventions that are often delivered at community level are usually more equitably distributed than those primarily delivered in fixed facilities or those that require changes in the home environment. Policy makers need to learn from community delivery channels to promote more equitable access to all RMNCH interventions.

**Funding:**

Bill & Melinda Gates Foundation and Wellcome Trust.

**Translations:**

For the French, Portuguese and Spanish translations of the abstract see Supplementary Materials section.

## Introduction

The UN's Millennium Development Goals (MDGs) did not prioritise equity when assessing a country's progress on women's and children's health, and no requirement was made for disaggregated monitoring indicators. A country was judged solely according to progress at national level towards the MDG targets.^1^ However, the issue of equity has come to orient the UN's Sustainable Development Goals, which reflect a commitment to “leave no one behind”.^2^ Reproductive, maternal, newborn, and child health (RMNCH) and nutrition has received wide attention and multiple coverage indicators are available for a large number of low-income and middle-income countries (LMICs) that often have high maternal and child mortality, undernutrition, and low coverage with essential interventions.^3^, ^4^, ^5^, ^6^

With increasing emphasis on within-country inequalities, in the past decade many reports have been published on equity by international agencies and initiatives, including WHO, UNICEF, and the Every Woman Every Child initiative, and many initiatives have updated their websites with information on inequalities in RMNCH, including the Countdown to 2030, Pan American Health Organization, and UNICEF.^7^, ^8^

**Research in context**

**Evidence before this study**

The evidence on socioeconomic inequalities in coverage of reproductive, maternal, newborn, and child health (RMNCH) indicators is ample, and in the past decade there was an increasing focus on within-country inequalities in reports by international agencies, multilateral organisations, and initiatives such as WHO, UNICEF, and the Every Woman Every Child initiative. Yet, to date, most studies have not systematically compared how coverage with essential interventions varies in terms of socioeconomic inequality across multiple countries. The 2003 *Lancet* Child Survival Series highlighted the importance of using appropriate delivery channels—namely, at the community level—to enable high and equitable coverage. A 2008 study by the Countdown to 2015 initiative found that skilled birth attendance and antenatal care, both of which are health facility-based interventions, had wider socioeconomic inequality than childhood immunisations, which are frequently provided at the community level. Similar findings were reported in a 2012 study by the same group. In these studies, a maximum of 11 interventions were compared in terms of inequality, with no a priori classification of delivery channels. In light of the Sustainable Development Goals' commitment to leave no one behind, information is urgently needed on how to reach the most vulnerable women and children.

**Added value of this study**

In our study we describe the magnitude of socioeconomic inequality in coverage with 20 RMNCH interventions in 36 countries from all world regions. This is the most comprehensive study on this topic to date. From a database of over 400 surveys done in over 110 countries, we systematically selected countries that had survey data for all 18 relevant preselected indicators, and two malaria-related indicators. We reanalysed raw data from all eligible countries using absolute and relative measures of inequality. Our study also expands on the concept of delivery channels to propose a framework with four main types: environmental, health facility-based, community-based, and culturally driven interventions. We found that our delivery channel classification coincided well with the median magnitude of inequalities favouring wealthier women, children, and households, which decreased progressively from environmental to culturally-driven interventions.

**Implications of all the available evidence**

We identified the potential of the delivery channels framework for designing health-sector and multisectoral approaches aimed at high and equitable RMNCH intervention coverage. The delivery channel framework is also relevant for monitoring and assessing the effect of health policies and programmes. Policy makers and health managers should learn from the success of community-based interventions when deciding how to scale and adapt these strategies to reduce inequalities in the coverage of environmental and health facility-based interventions. Reducing inequalities in RMNCH intervention coverage is more urgent now than ever in the context of the COVID-19 pandemic, which is disproportionately affecting the poorest subgroups of the population.

Socioeconomic inequalities in RMNCH coverage indicators have been widely studied, yet few systematic multicountry studies exist. In the 2003 *Lancet* Child Survival Series, the importance of using appropriate delivery channels, particularly those at community level, was highlighted as essential for reaching high and equitable coverage.^9^ Later publications by the Countdown to 2015 group provided further evidence that indicators delivered at community level were usually delivered more equitably among socioeconomic groups than those requiring access to fixed health facilities.^3^, ^4^

By examining the list of available RMNCH coverage indicators that are often measured in national surveys, we classified interventions into four main groups: environmental, primarily health facility-based, primarily community-based, and culturally driven interventions. We aimed to systematically compare coverage of different key RMNCH indicators in terms of inequality in LMICs, using the delivery channel framework.

## Methods

### Data sources

In this cross-sectional study, we analysed publicly available data from the Demographic Health Surveys (DHS) and the Multiple Indicator Cluster Surveys (MICS). The DHS and MICS are nationally representative cross-sectional surveys, mostly done in LMICs, that contain information on household characteristics, reproductive health, women's and children's health, nutrition, and mortality. The surveys use similar multi-stage cluster sampling methods to select women of reproductive age (15–49 years) and children younger than 5 years for inclusion, so making them highly comparable.^10^, ^11^

The International Center for Equity in Health (ICEH)/Countdown database includes over 400 surveys from more than 110 countries. We used the most recent survey since 2010 up to the end of 2019 from each country that contained data on all of our 18 preselected RMNCH indicators because several indicators (eg, postnatal care) were not available from earlier surveys. Because of low availability of adolescent health indicators, we were unable to include them in our analyses. We excluded surveys that did not provide information on asset indices and the few surveys from high-income countries.

All DHS and MICS were done by national central statistics agencies or research institutes. The institutions that approved, provided funding for, or implemented the surveys were responsible for ethical clearance, which guaranteed informed consent (for children, consent was given by their caregiver) and confidentiality of respondents' information.

### Selection of indicators

For our analyses, we selected 18 key internationally agreed on indicators for intervention coverage, plus two malaria-related indicators from countries with available information. The indicators describe essential interventions along the continuum of care for women, neonates, and children, including family planning; institutional childbirth; prenatal and postnatal care; immunisations; case management for common illnesses among neonates and children; infant and child feeding; the household environment; and two malaria-related interventions. We reanalysed data for the indicators by dividing the number of women, children, and households who received the intervention or used the health service by the target population or the number of women, children or households in need of the intervention or health service, in accordance with the standard definitions provided by international agencies and monitored by the Countdown to 2030 initiative.^8^ The 20 indicators we studied were included for monitoring by the Countdown initiative because of their relevance in the scientific literature pertaining to RMNCH globally, and, to this end, for their importance as key interventions along the continuum of care. We recalculated indicators from the original survey data from the ICEH database, and all calculations considered the complex survey design, including weighting and cluster sampling. We compared the results obtained with official reports and the results were consistent. The indicators selected, their definitions, and the target age groups are in appendix 4 (pp 7–8).

On the basis of how interventions are usually delivered to women, children, and households in most LMICs,^1^, ^3^, ^4^, ^9^ we grouped the 20 interventions into four groups, as follows: environmental interventions, including clean fuels for cooking, improved sanitation, and piped water; health facility-based interventions, including antenatal care (four or more visits), institutional childbirth, postnatal visit for mothers, and birth registration; community-based interventions, including demand for family planning satisfied with modern methods (appendix 4 p 7), postnatal visit for babies, diphtheria-tetanus-pertussis (DTP3) immunisation, measles immunisation, polio immunisation, vitamin A supplementation, care seeking for any disease, oral rehydration treatment for diarrhoea, minimum dietary diversity, antimalarial treatment for fever, and ownership of insecticide-treated bednets or household sprayed; and culturally driven interventions, including exclusive breastfeeding (0–5 months) and continued breastfeeding (12–15 months). Of these interventions, antimalarial treatment for fever and ownership of insecticide-treated bednets or household sprayed were only collected in countries endemic for malaria.

### Statistical analysis

To measure the magnitude of inequality in the indicators studied, we used the information available in the DHS and MICS datasets. Households' socioeconomic positions were derived using asset indices based on household possessions (eg, television and stove) and characteristics (eg, presence of electricity and other utilities and type of toilet) among other variables.^12^, ^13^ Because of the different relevance of specific assets in urban and rural settings, DHS and MICS use two separate principal component analyses in each area and combine them into a single score for each country using a scaling procedure that permits comparability between urban and rural households. Finally, we split the score into quintiles, weighted by the number of members in each household.^14^ We used values of the asset index provided by DHS and MICS in each dataset.

We measured inequality using two summary indices, the slope index of inequality (SII) and the concentration index (CIX). We calculated the SII using a logistic regression model that uses the natural log of the odds of the dependent variable—in our case, intervention coverage—to create a continuous criterion on which linear regression is performed with the assets index as the explanatory variable. This approach permits calculation of the percentage point difference between adjusted values of the health indicator being studied for the top and bottom extremes of the wealth distribution, while considering the entire distribution of coverage according to wealth. SII values range from −100 to 100 percentage points and positive values indicate higher coverage among the wealthy (pro-rich pattern), while negative values indicate higher coverage among the poor (pro-poor pattern). The CIX is based on a similar concept to the Gini index for the concentration of wealth, describing the distance between an observed distribution and total equality. It ranks individuals according to socioeconomic position on the x-axis and plots cumulative intervention coverage on the y-axis. A value of zero means that there is no inequality. Like the SII, positive values for the CIX show pro-rich inequality (ie, interventions that have higher coverage among wealthy women, children, or households than among poor women, children, or households) and negative values indicate pro-poor inequality (ie, interventions that have higher coverage among poor women, children, or households than among wealthy women, children, or households). The SII reflects absolute inequality (a measure reflecting differences), whereas the CIX shows relative inequality (or ratios).^15^, ^16^ We calculated SEs for the SII and CIX to estimate 95% CIs. We calculated median values for the SII and CIX with IQRs. We also calculated mean SII and CIX with their 95% CIs.

For obtaining pooled estimates of the summary inequality measures, we calculated the mean (SD), range, and median (IQR) for each combination of intervention and summary measure, using countries as the units of analyses. Given the relatively small number of countries included in our analyses and the uneven representation of all world regions and income groups, we opted to give equal weights to all countries and, in presenting graphical results, we focused on median values. We stratified our analyses by World Bank country income groups, according to how countries were classified in 2015, which was the median year of the surveys included in the analyses.^17^ We compared mean values of the slope and concentration indices among interventions in the four delivery channels using variance-weighted least squares regression. We calculated p values using an F test derived from variance-weighted least squares regression.

To test the robustness of our findings, we did two sensitivity analyses. First, we replicated the main analyses restricted to the 21 malaria-endemic countries that had information on all 20 indicators, which allowed comparison of inequality levels in the 18 non-malaria indicators with the two malaria-related indicators in the same countries. Second, we replicated the main analyses using the 104 countries in the Countdown database that had survey data for 2010–19 and that provided information on any of the 20 indicators included in the main analyses. In this second analysis we explored whether restricting our main analysis to the 36 countries with full information might have introduced bias in our results.

We considered p values of less than 0·05 to be significant. We did all analyses using Stata (version 16.1) and handled all databases using Microsoft Excel spreadsheets (Microsoft, WA, USA).

### Role of the funding source

The funders of the study had no role in study design, data collection, data analysis, data interpretation, or writing of the report.

## Results

We identified 36 eligible surveys for the study period (2010–19) from low-income and middle-income countries (appendix 4 p 9). We excluded two countries because they did not have information on household socioeconomic position (Cuba in 2010 and 2014, and Qatar in 2012), and another 63 countries had surveys in this period that did not contain all 20 continuum of care indicators. Most often, indicators that were unavailable in the surveys included vitamin A supplementation, care seeking for any illness, and postnatal care for women and neonates. The two malaria indicators were only available for the 21 countries where this disease is endemic.

The 36 surveys with information on the required indicators included 12 surveys done in west and central Africa, ten in eastern and southern Africa, one in the Middle East and north Africa, one in eastern Europe and central Asia, five in south Asia, three in east Asia and the Pacific, and four in Latin America and the Caribbean (appendix 4 p 9).

**Figure 1:**
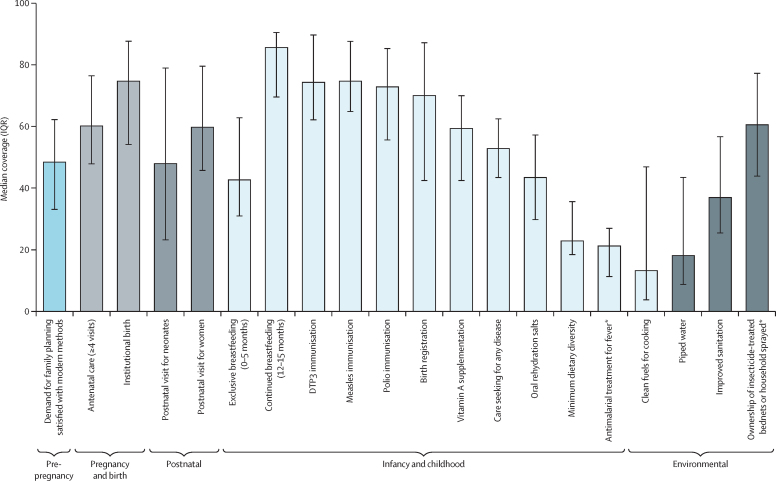
Median coverage of 20 RMNCH interventions in 36 LMICs, by reproductive or childhood phase and environmental interventions, 2010–19 Bars show median coverage, with error bars showing IQRs. DTP3=diphtheria-tetanus-pertussis. LMICs=low-income and middle-income countries. RMNCH=reproductive, maternal, newborn, and child health. *Based on 21 countries with endemic malaria.

Median coverage of the 20 RMNCH indicators studied are shown in figure 1, with 18 indicators identified in 36 countries and two additional malaria-related interventions in 21 countries where this disease is endemic. Median coverage levels were above 70% for continued breastfeeding at 12–15 months, childhood immunisations, birth registration, and institutional birth, whereas minimum dietary diversity, antimalarial treatment for fever, piped water, and clean fuels for cooking all had a median coverage below 25%. Mean coverage values and their 95% CIs are in appendix 4 (p 12).

Patterns of coverage of the 20 indicators in LMICs are similar (appendix 4 p 16), but coverage for most indicators was slightly lower in low-income countries than in middle-income countries, particularly for use of clean fuels. The exception is continued breastfeeding, for which low-income countries had higher coverage than middle-income countries. Similar patterns were observed in the coverage of the 20 indicators across the 21 countries with endemic malaria as across all 36 included countries (appendix 4 pp 13–15, 17). In countries that are endemic for malaria, median coverage was above 66% in continued breastfeeding at 12–15 months, childhood immunisations, birth registration, and institutional birth, whereas median coverage was below 22% for minimum dietary diversity, antimalarial treatment for fever, piped water, and clean fuels for cooking.

**Figure 2:**
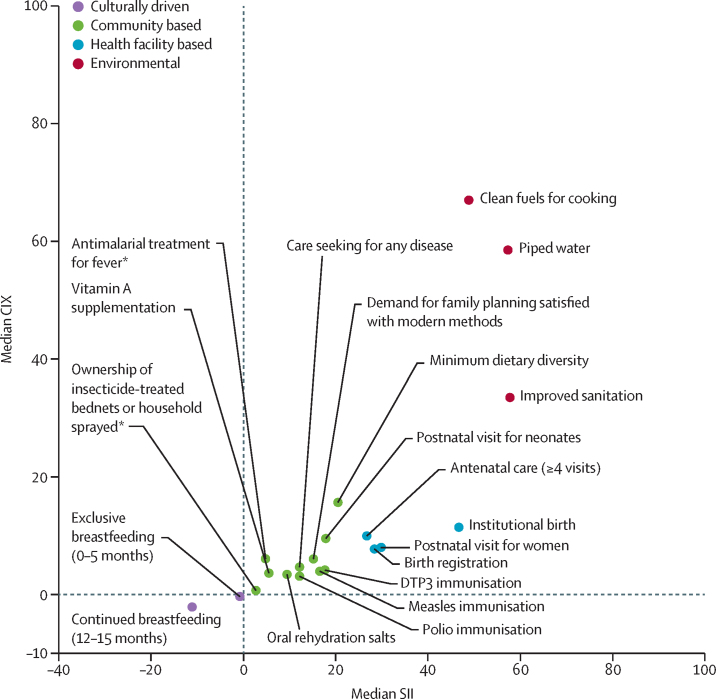
Median values for absolute (SII) and relative (CIX) socioeconomic inequalities in coverage of 20 RMNCH interventions in 36 LMICs, 2010–19 Datapoints are median values for each intervention. Full data are in appendix 4 (p 12). CIX=concentration index. DTP3=diphtheria-tetanus-pertussis. LMICs=low-income and middle-income countries. RMNCH=reproductive, maternal, newborn, and child health. SII=slope index of inequality. *Based on 21 countries with endemic malaria.

Across RMNCH indicators, environmental interventions were the most unequal in terms of absolute and relative socioeconomic inequalities, particularly use of clean fuels, which had median levels of SII of 48·8 (8·6–85·7) and CIX of 67·0 (45·0–85·8; figure 2; appendix 4 pp 14–15). Interventions primarily delivered in health facilities—namely, institutional birth (median SII 46·7 [23·1–63·3] and CIX 11·4 [4·5–23·4]) and antenatal care (median SII 26·7 [17·0–47·2] and CIX 10·0 [4·2–17·1])—also show substantial pro-rich patterns. By comparison, primarily community-based interventions were more equitably distributed—eg, oral rehydration salts (median SII 9·4 [2·9–19·0] and CIX 3·4 [1·3–8·5]) and polio immunisation (SII 12·1 [2·3–25·0] and CIX 3·1 [0·5–7·1]). Among community-based interventions, minimum dietary diversity had the largest SII and CIX values (figure 2). Culturally driven indicators (ie, exclusive breastfeeding from 0–5 months and continued breastfeeding at 12–15 months) had pro-poor inequality. Weighted least squares regression confirmed that the magnitude of inequalities according to both SII (p=0·0052) and CIX (p=0·0048) varied significantly among the four categories of delivery channels (table).

**Table:** Variance-weighted least squares regression with the summary indices of inequality as outcomes, according to RMNCH delivery channel

	**Mean coverage (SE; 95% CI)**	**p value**
Slope index of inequality*		
Environmental	62·5 (20·2; 22·8 to 102·1)	0·0020
Health facility based	41·5 (15·2; 11·7 to 71·3)	0·0063
Community based	23·1 (12·4;–1·2 to 47·3)	0·062
Culturally driven	0 (ref)	..
**Concentration index**†		
Environmental	47·8 (15·3; 17·8 to 77·8)	0·0018
Health facility based	15·5 (6·6; 2·5 to 28·6)	0·020
Community based	9·1 (4·9;–0·6 to 18·7)	0·065
Culturally driven	0 (ref)	..

RMNCH=reproductive, maternal, newborn, and child health.

* Overall p=0·0052.

† Overall p=0·0048.

**Figure 3:**
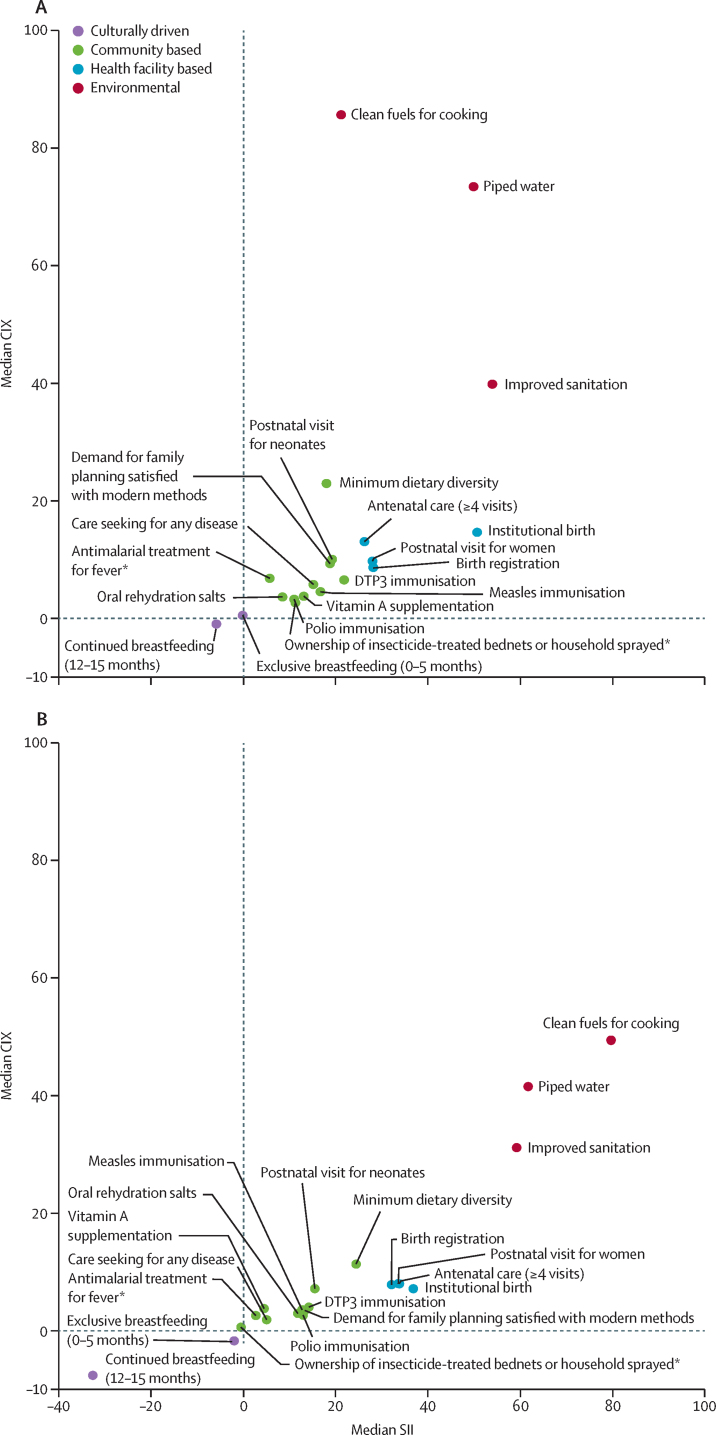
Median values for absolute (SII) and relative (CIX) socioeconomic inequalities in coverage of 20 RMNCH interventions in 17 low-income countries (A) and 19 middle-income countries (B), 2010–19 Datapoints show median values for each intervention. CIX=concentration index. DTP3=diphtheria-tetanus-pertussis. RMNCH=reproductive, maternal, newborn, and child health. SII=slope index of inequality. *Based on 21 countries with endemic malaria.

Although general patterns in inequality for LMICs are similar, relative (but not absolute) inequalities were mostly smaller in middle-income countries than in low-income countries (figure 3; appendix 4 p 13). The most substantial differences were observed for clean fuels for cooking and piped water, for which the CIX was much higher in low-income countries than in middle-income countries, and continued breastfeeding for which absolute, pro-poor inequalities were much greater in middle-income countries than in low-income countries.

When examining SII and CIX values of the 20 indicators in the 21 countries with endemic malaria (appendix 4 pp 5, 14), among environmental indicators, clean fuels for cooking had the lowest absolute inequality, less than all health facility-based interventions, and the largest relative inequality. DTP3 and measles immunisations had the highest absolute inequality of all the community-based interventions, while minimum dietary diversity had the greatest relative inequality. Exclusive breastfeeding presented slight pro-rich inequality.

In our sensitivity analysis using all available datapoints from the most recent survey since 2010 for 104 countries, we observed the same patterns as in our main analyses, with relative inequalities for interventions and absolute inequalities for facility-based interventions being smaller for all 104 countries than for the 36 countries with full information (appendix 4 p 6).

## Discussion

Across 36 LMICs, including 21 malaria-endemic countries, coverage levels of RMNCH indicators were highest for continued breastfeeding at 12–15 months, childhood immunisations, birth registration, and institutional birth, and lowest for minimum dietary diversity, antimalarial treatment for fever, piped water, and clean fuels for cooking. High-level aggregation of levels of coverage and the magnitude of inequalities is useful for providing a global view on such indicators. Country-by-country disaggregation of results, which will support decision making at national level, is available on the Countdown to 2030 website where national coverage and equity profiles are available using the same database used in these analyses.

Pro-rich inequalities were found for most indicators, with substantial clustering of inequality patterns according to the four groups of delivery channels. Interventions delivered in the household environment had the highest values of the slope and concentration indices, indicating greater coverage in wealthy households than in poor households. One reason for this gap in coverage is probably because of the cost of housing and water and sanitation facilities, which might be regarded as supra-individual, multisectoral interventions because they require access to a network in the community, limiting their availability to the poorest families in hard-to-reach areas.^18^ A caveat in these analyses is that the asset indices we used to classify families into wealth quintiles include some variables related to housing and access to water and sanitation, creating a positive feedback loop in which these indicators would inevitably be associated with wealth. Nevertheless, a comprehensive set of analyses by UNICEF found that, even when environmental indicators are not included in the asset index, they remain highly unequally distributed.^19^

Interventions in health facilities were also highly inequitably distributed. These interventions include antenatal and postnatal care and institutional childbirth. Inequality in delivery of these interventions is driven by geographical (distance from fixed facilities) and cultural barriers, mistreatment of women in facilities,^20^ and costs incurred by paying for services (in many countries) and transportation.^21^ Birth registration was also classified in this group because it requires access to vital record facilities, often with costs incurred.^22^

Community-based interventions had the least pro-rich inequalities. In many countries, these interventions are delivered by community health workers at little or no cost to families, including immunisations, case management for common diseases (malaria, diarrhoea, and pneumonia), and insecticide-treated bednets or household spraying with insecticides. The group of interventions also includes family planning services, which might be provided at community level, in health facilities, or a combination of both, depending on the country. Minimum dietary diversity was included in this group because it is also often part of nutrition counselling at community level, although this intervention is more inequitable than other community interventions because it also requires families to purchase a variety of foods, some of which might be expensive (eg, animal-based, protein-rich foods).^23^

The culturally driven interventions pertained to infant and young child feeding: exclusive breastfeeding up to age 6 months, and continued breastfeeding at age 12–15 months. Continued breastfeeding had pro-poor absolute inequality, as measured by the SII. For exclusive breastfeeding up to age 6 months, we found almost no inequality when countries were pooled. These results are similar to those of a 2016 comprehensive review.^24^ We refer to these behaviours as culturally driven because they are not substantially affected by health services, being determined by culture and economics (eg, affordability of non-breastmilk).^25^

In low-income countries, clean fuels for cooking had a smaller SII value than all health facility-based interventions, and continued breastfeeding had substantially larger pro-poor absolute inequalities in middle-income countries than in low-income countries. All three environmental indicators had less relative inequality in middle-income countries than in low-income countries.

When comparing values of absolute and relative inequality across countries, it is important to be aware of their association with intervention coverage because absolute inequalities are usually lowest when coverage approaches 0% and 100%, whereas these inequalities are usually highest when overall coverage is around 50%.^26^ However, relative inequality is strongly influenced by low coverage among the most disadvantaged sectors of the population, and decreases as coverage increases.^26^ In the case of environmental interventions, particularly clean fuels for cooking, the low level of coverage in low-income countries corresponds with a low SII value and a high CIX value, whereas the higher coverage for childhood immunisations in middle-income countries than in low-income countries lead to low SII values.

Our findings support and expand on those of previous studies that found that health facility interventions usually have more socioeconomic inequality than community-delivered interventions.^3^, ^4^ In addition to these two groups of interventions that have been recognised in the past, we identified two additional categories of interventions—environmental and culturally driven—that were situated in the extremes of the inequality spectrum.^27^

Our analyses have limitations. First, of over 100 countries with surveys in the ICEH/Countdown database, only 36 had information on all 18 key indicators, and 21 on malaria interventions. To compare summary indicators of inequality across countries, we needed to have information on all indicators, hence the restriction to 36 countries with data. In our sensitivity analysis of 21 countries with endemic malaria, we aimed to compare inequalities in the two malaria indicators with those in the remaining 18 indicators, while also ensuring that the comparison was limited to countries with the full set of indicators. Notably, our country-level analyses do not account for subnational geographical inequalities in coverage, which are probably present for many interventions.^27^, ^28^ A second limitation is that our classification of delivery channels is somewhat arbitrary. We are not aware of any systematic or objective review of which channels are used in the majority of LMICs. Additionally, several interventions, particularly those in the community-based category, are also likely to be delivered at the facility level for children whose families have access to such services (eg, family planning). Therefore, the lower level of inequality for these interventions than for the other interventions in more strict categories is indicative of a mixture of community delivery for poor children and facility delivery for those from wealthier families. The mixture of facility-based and community-based delivery systems is probably the case for immunisations and case management of common childhood illnesses. However, interventions such as institutional childbirth that cannot be provided outside of health facilities were unequally distributed. A third limitation was that our assessment of wealth was based on a relative scale; the poorest quintile in a given country might be wealthier than the second or third quintile in another. Nevertheless, the equity literature shows that relative poverty might be just as important as absolute poverty in terms of predicting deprivation^29^ and health status.^30^

The scientific literature on intervention coverage along the continuum of care highlights the importance of adolescent health. We did not include any interventions for adolescents in our analyses because few of these are included in the surveys that were analysed, particularly for adolescent boys. Although a separate study of family planning and antenatal and delivery care interventions is possible for adolescent girls,^28^ sample sizes are smaller than for all women of reproductive age, and we opted to provide results for this wider age range. Hopefully, inclusion of adolescent health indicators in future surveys will allow the expansion of the present analyses.

Our study also has several strengths. First, we reanalysed raw data from all countries studied using both absolute and relative measures of inequality. Second, through a systematic approach to the selection of countries with all relevant indicators based on a database of over 400 surveys in over 110 countries, we included countries from all world regions, although more than 50% of countries in our main analyses were in Africa because of the higher frequency with which surveys are done on the continent than in other regions. Finally, the aforementioned factors allow for our work to provide a comprehensive overview of coverage along the continuum of care.

Although publications in scientific journals usually initially reach global health audiences rather than country-level policy makers or health managers, the *Lancet* Series on Child Survival, Neonatal Survival, and Maternal Health show the importance of scientific publications as early drivers of action that eventually take place at country level. These Series papers were behind the creation of the Countdown to 2015 initiative (now Countdown to 2030) and the Partnership for Maternal, Newborn and Child Health, which among other initiatives have catalysed resources and promoted accountability at global and national levels. Explicit incorporation of RMNCH delivery channels in national planning and monitoring initiatives is essential. Such a framework will be useful in designing multisectoral approaches to the reduction of health inequalities.^31^ Policy makers need to learn from the successes of community-based interventions to scale and adapt these strategies towards reducing inequalities in the coverage of environmental, health facility-based, and culturally driven interventions.

## Data sharing

Individual-level data used in these analyses are publicly available on the DHS and MICS websites. The processed database with coverage levels and summary indices of inequality are available on the WHO Health Equity Monitor database, which is populated with data analysed at the ICEH at the Federal University of Pelotas.

## Declaration of interests

We declare no competing interests.

## Supplementary Materials

French translation of the abstract

Portuguese translation of the abstract

Spanish translation of the abstract
