# Supplementary material for: Delivery channels and socioeconomic inequalities in coverage of reproductive, maternal, newborn, and child health interventions: analysis of 36 cross-sectional surveys in low-income and middle-income countries
Source: Lancet Glob Health. 2021 May 26;9(8):e1101–9. doi: 10.1016/S2214-109X(21)00204-7 (PMC8295042; doi:10.1016/S2214-109X(21)00204-7)
Supplement: French translation of the abstract [file mmc1.pdf]

THE LANCET
Global Health

Supplementary appendix 1

This translation in French was submitted by the authors and we reproduce it as supplied. It has not been peer reviewed. *The Lancet*'s editorial processes have only been applied to the original in English, which should serve as reference for this manuscript.

Cette traduction en français a été proposée par les auteurs et nous l'avons reproduite telle quelle. Elle n'a pas été examinée par des pairs. Les processus éditoriaux du *Lancet* n'ont été appliqués qu'à l'original en anglais et c'est cette version qui doit servir de référence pour ce manuscrit.

Supplement to: Leventhal DGP, Crochemore-Silva I, Vidaletti LP, Armenta-Paulino N, Barros AJD, Victora CG. Delivery channels and socioeconomic inequalities in coverage of reproductive, maternal, newborn, and child health interventions: analysis of 36 cross-sectional surveys in low-income and middle-income countries. *Lancet Glob Health* 2021; published online May 26. http://dx.doi.org/10.1016/S2214-109X(21)00204-7.

Canaux de prestation et inégalités socio-économiques dans la couverture des interventions de santé reproductive, maternelle, néonatale et infantile : une analyse de 36 enquêtes transversales dans des pays à faible revenu et à revenu intermédiaire

Daniel G P Leventhal, Inácio Crochemore-Silva, Luis P Vidaletti, Nancy Armenta-Paulino, Aluísio J D Barros, Cesar G Victora

Sommaire

Contexte Les inégalités dans la couverture des interventions de santé reproductive, maternelle, néonatale et infantile (SRMNI) sont décrites dans les rapports internationaux, cependant, on en sait peu sur la façon dont les inégalités socio-économiques dans la couverture des interventions varient dans les pays à faible revenu et à revenu intermédiaire (PRFI). Notre objectif est de systématiquement comparer la couverture des interventions clés SRMNI en termes de l'inégalité dans des PRFI, en utilisant le cadre de canaux de prestation.

Méthodes Dans cette étude transversale, nous avons identifié des enquêtes démographiques et sanitaires (DHS) et des enquêtes en grappes à indicateurs multiples (MICS) venant des PRFI et accessibles au public, contenant des informations sur les caractéristiques des ménages, la santé reproductive, la santé des femmes et des enfants, la nutrition et la mortalité. Nous avons identifié l'enquête la plus récente de la période 2010–19 pour 36 pays qui contenaient des données pour un ensemble présélectionné de 18 indicateurs d'interventions. Vingt et un pays avaient également des informations sur deux interventions antipaludiques courantes. Nous avons classé les interventions en quatre groupes en fonction de leurs canaux de prestation prédominants : interventions basées dans les établissements de santé, interventions communautaires, interventions au niveau de l'environnement domestique et celles déterminées par la culture (y compris les pratiques d'allaitement). Dans chaque pays, les quintiles de richesse sont dérivés des informations sur les indices de biens des ménages. Nous avons étudié deux mesures récapitulatives des inégalités socio-économiques à l'intérieur d'un pays : les inégalités absolues (semblables aux différences de couverture entre les femmes et les enfants des ménages riches et pauvres) en utilisant « the slope index of inequality (SII) » et les inégalités relatives (semblables au ratio des niveaux de couverture pour les femmes et les enfants riches et pauvres) en utilisant l'indice de concentration (CIX). Nous considérons les inégalités en faveur des pauvres (pro-pauvres) lorsque la couverture des interventions diminue avec l'augmentation de la richesse des ménages, et les inégalités en faveur des riches (pro-riches) lorsque la couverture des interventions augmente à mesure que la richesse des ménages augmente.

Résultats Entre 36 PRFI inclus dans nos analyses, la couverture de la plupart des interventions avait des modèles pro-riches dans la plupart des pays, à l'exception de deux indicateurs de l'allaitement maternel, qui avaient principalement une couverture plus élevée parmi les femmes et les enfants pauvres que les femmes et les enfants riches. Les interventions environnementales étaient les plus inégales, en particulier l'utilisation de combustible propre, avec des niveaux médians de SII de 48 · 8 (8 · 6–85 · 7) et de CIX de 67 · 0 (45 · 0–85 · 8). Les interventions principalement dispensées dans les établissements de santé, à savoir l'accouchement institutionnel (SII médian 46 · 7 [23 · 1–63 · 3] et CIX 11 · 4 [4 · 5–23 · 4]) et les soins prénatals (SII médian de 26 · 7 [17 · 0–47 · 2] et CIX de 10 · 0 [4 · 2–17 · 1]) avaient aussi généralement des modèles pro-riches. En comparaison, les interventions principalement communautaires, y compris celles contre le paludisme, étaient plus équitablement réparties, par exemple, des sels de réhydratation orale (SII médian 9 · 4 [2 · 9–19 · 0] et CIX 3 · 4 [1 · 3–25 · 0]) et la vaccination contre la polio (SII 12 · 1 [2 · 3–25 · 0] et CIX 3 · 1 [0 · 5–7 · 1]). Les différences entre les quatre types de canaux de distribution en termes des deux indices d'inégalité étaient significatives (SII p = 0 · 0052 ; CIX p = 0 · 0048).

Interprétation Les interventions régulièrement fournies au niveau communautaire sont généralement plus équitablement réparties que celles qui sont principalement fournies dans des installations fixes ou celles qui nécessitent des changements dans l'environnement domestique. Les décideurs en politiques publiques doivent comprendre le rôle des canaux de prestation communautaires pour promouvoir un accès plus équitable à toutes les interventions SRMNI.

Financement Fondation Bill & Melinda Gates et Wellcome Trust

Copyright © 2021 Auteur (es). Publié par Elsevier Ltd. Il s'agit d'un article de libre accés sous la licence CC BY 4.0.
