# Supplementary material for: Delivery channels and socioeconomic inequalities in coverage of reproductive, maternal, newborn, and child health interventions: analysis of 36 cross-sectional surveys in low-income and middle-income countries
Source: Lancet Glob Health. 2021 May 26;9(8):e1101–9. doi: 10.1016/S2214-109X(21)00204-7 (PMC8295042; doi:10.1016/S2214-109X(21)00204-7)
Supplement: Portuguese translation of the abstract [file mmc2.pdf]

THE LANCET
Global Health

Supplementary appendix 2

This translation in Portuguese was submitted by the authors and we reproduce it as supplied. It has not been peer reviewed. The Lancet's editorial processes have only been applied to the original in English, which should serve as reference for this manuscript.

Esta tradução em português foi submetida pelos autores e nós não fizemos quaisquer alterações. Esta versão não foi revista por pares. O processo editorial do The Lancet só foi aplicado à versão original em inglês, que deve servir como referência para este artigo.

Supplement to: Leventhal DGP, Crochemore-Silva I, Vidaletti LP, Armenta-Paulino N, Barros AJD, Victora CG. Delivery channels and socioeconomic inequalities in coverage of reproductive, maternal, newborn, and child health interventions: analysis of 36 cross-sectional surveys in low-income and middle-income countries. *Lancet Glob Health* 2021; published online May 26. http://dx.doi.org/10.1016/S2214-109X(21)00204-7.

Canais de prestação e desigualdades socioeconômicas na cobertura de intervenções de saúde reprodutiva, materna, neonatal e da criança: uma análise de 36 inquéritos transversais em países de baixa e média renda.

Daniel G P Leventhal, Inácio Crochemore-Silva, Luis P Vidaletti, Nancy Armenta-Paulino, Aluísio J D Barros, Cesar G Victora

Resumo

Contexto Desigualdades na cobertura de intervenções para a saúde reprodutiva, materna, neonatal e da criança (SRMNC) têm sido descritas em vários relatórios internacionais, mas pouco se sabe sobre como desigualdades socioeconômicas na cobertura de intervenções variam entre vários países de renda baixa e média (PRBMs). O objetivou desse estudo foi comparar sistematicamente a cobertura de intervenções SRMNC chaves em termos de desigualdade em PRBMs, utilizando o marco de canais de prestação.

Métodos Neste estudo transversal, nós identificamos Inquéritos Demográficos e de Saúde (DHS) e Inquéritos de Indicadores Múltiplos (MICS) publicamente disponíveis de PRBMs, contendo informações sobre características dos domicílios, saúde reprodutiva, saúde de mulheres e de crianças, nutrição e mortalidade. Identificamos os inquéritos mais recentes do período entre 2010 e 2019 para 36 países que apresentavam dados para um conjunto pré-selecionado de 18 indicadores de intervenções. Vinte e um países também tiveram informações sobre duas intervenções comuns para a malária. Classificamos as intervenções em quatro grupos de acordo com os seus canais de prestação predominantes: intervenções baseadas em unidades sanitárias, prestadas em nível comunitário, intervenções de níveis ambientais e aquelas determinadas pela cultura (incluindo práticas de amamentação). Dentro de cada país, quintis de riqueza foram derivados de informações sobre índices de bens domiciliares. Utilizamos duas medidas sumárias de desigualdade socioeconômica dentro dos países: desigualdades absolutas (semelhantes a diferenças de cobertura entre crianças de domicílios ricos e pobres) usando o índice angular de desigualdade (IAD), e desigualdades relativas (semelhantes à razão de níveis de cobertura para crianças ricas e pobres) utilizando o índice de concentração (IC). Consideramos desigualdades pró-pobres quando a cobertura de intervenções diminuiu conforme aumento da riqueza dos domicílios, e desigualdades pró-ricos quando a cobertura de intervenções aumentou conforme aumento da riqueza domiciliar.

Achados Entre os 36 PRBMs incluídos na nossa análise, a cobertura da maioria das intervenções teve padrões pró-ricos na maioria dos países, com a exceção de dois indicadores de amamentação, que tiveram majoritariamente a cobertura mais alta entre mulheres e crianças pobres do que mulheres e crianças ricas. As intervenções ambientais foram as mais desiguais,

particularmente uso de combustível limpo, que teve medianas do IAD de 48.8 (8.6-85.7) e do IC de 67.0 (45.0-85.8). As intervenções principalmente prestadas em unidades sanitárias – a saber, parto institucional (mediana do IAD 46.7 [23.1-63.3] e IC 11.4 [4.5-23.4]) e cuidados pré-natais (mediana do IAD 26.7 [17.0-47.2] e IC 10.0 [4.2-17.1]) – também tiveram padrões pró-ricos em geral. Em comparação, intervenções principalmente prestadas na comunidade, incluindo aquelas contra a malária, foram distribuídas de maneira mais equitativa – ex. sais de hidratação oral (mediana do IAD 9.4 [2.9-19.0] e IC 3.4 [1.3-25.0]) e imunização contra poliomielite (IAD 12.1 [2.3-25.0] e IC 3.1 [0.5-7.1]). Diferenças entre os quatro tipos de canais de prestação em termos de ambos os índices de desigualdade foram significativas (IAD p=0.0052; IC p=0.0048).

Interpretação As intervenções frequentemente prestadas no nível comunitário são geralmente distribuídas de maneira mais equitativa do que aquelas principalmente prestadas em unidades sanitárias fixas, ou aquelas que exigem mudanças no ambiente domiciliar. Formuladores de políticas públicas precisam compreender o papel dos canais de prestação comunitários para promover o acesso mais equitativo a todas as intervenções SRMNC.

Financiamento Bill and Melinda Gates Foundation e Wellcome Trust

Copyright © 2021 O(s) Autor(es). Publicado por Elsevier Ltd. Este é um artigo de Acesso Aberto sob a licença CC BY 4.0.
