# Supplementary material for: Delivery channels and socioeconomic inequalities in coverage of reproductive, maternal, newborn, and child health interventions: analysis of 36 cross-sectional surveys in low-income and middle-income countries
Source: Lancet Glob Health. 2021 May 26;9(8):e1101–9. doi: 10.1016/S2214-109X(21)00204-7 (PMC8295042; doi:10.1016/S2214-109X(21)00204-7)
Supplement: Spanish translation of the abstract [file mmc3.pdf]

THE LANCET
Global Health

## Supplementary appendix 3

This translation in Spanish was submitted by the authors and we reproduce it as supplied. It has not been peer reviewed. *The Lancet*'s editorial processes have only been applied to the original in English, which should serve as reference for this manuscript.

Los autores nos proporcionaron esta traducción al español y la reproducimos tal como nos fue entregada. No la hemos revisado. Los procesos editoriales de *The Lancet* se han aplicado únicamente al original en inglés, que debe servir de referencia para este manuscrito.

Supplement to: Leventhal DGP, Crochemore-Silva I, Vidaletti LP, Armenta-Paulino N, Barros AJD, Victora CG. Delivery channels and socioeconomic inequalities in coverage of reproductive, maternal, newborn, and child health interventions: analysis of 36 cross-sectional surveys in low-income and middle-income countries. *Lancet Glob Health* 2021; published online May 26. http://dx.doi.org/10.1016/S2214-109X(21)00204-7.

# Canales de prestación y desigualdades socioeconómicas en la cobertura de intervenciones en salud reproductiva, materna, neonatal y de niños: Análisis de 36 encuestas transversales en países de ingresos medianos y bajos

*Daniel G P Leventhal, Inácio Crochemore-Silva, Luis P Vidaletti, Nancy Armenta-Paulino, Aluísio J D Barros, Cesar G Victora*

## Resumen

**Antecedentes.** Los informes internacionales se han enfocado en describir las desigualdades en la cobertura de intervenciones en salud reproductiva, materna, neonatal y de niños (SRMNN), pero poco se sabe acerca de cómo las desigualdades socioeconómicas en la cobertura de intervenciones varían entre los países de ingresos medianos y bajos. El objetivo fue comparar sistemáticamente la cobertura de intervenciones de SRMNN claves en términos de desigualdad en países de ingresos medianos y bajos, utilizando el marco de canales de prestación.

**Métodos.** En este estudio transversal, identificamos las Encuestas Demográficas y de Salud (DHS) y Encuestas de Indicadores Múltiples por Conglomerados (MICS) públicas y disponibles para países de ingresos medianos y bajos con información sobre características de los hogares, salud reproductiva, salud de mujeres y niños, nutrición y mortalidad. Seleccionamos las encuestas más recientes entre 2010 y 2019 para 36 países con datos para un conjunto preseleccionado de 18 intervenciones. Veinte y uno países también tuvieron informaciones sobre dos intervenciones comunes para la malaria. Clasificamos las intervenciones en cuatro grupos de acuerdo con sus principales canales de prestación: intervenciones basadas en unidades sanitarias, en la comunidad, ambientales y aquellas determinadas por la cultura (incluyendo prácticas de lactancia). Dentro de cada país, obtuvimos los quintiles de riqueza a partir de la información del índice de bienes del hogar. Analizamos dos medidas resumen de desigualdad socioeconómica dentro de los países: absolutas (diferencias de cobertura entre las mujeres y niños de hogares ricos y pobres) usando el índice de desigualdad de la pendiente (SII), y relativas (ratios de cobertura para mujeres y niños ricos y pobres) utilizando el índice de concentración (CIX). Desigualdades pro-pobres están presentes cuando la cobertura de intervenciones se reduce conforme aumenta la riqueza de los hogares, y pro-ricos cuando la cobertura aumenta conforme aumenta la riqueza del hogar.

**Hallazgos.** En los 36 países de ingresos medianos y bajos incluidos en nuestro análisis, la cobertura de la mayoría de las intervenciones tuvo patrones pro-ricos para la mayoría de los países, con excepción de dos indicadores de lactancia, que tuvieron, mayormente cobertura más alta entre mujeres y niños pobres que en los ricos. Las intervenciones ambientales fueron las más desiguales, particularmente, el uso de combustible limpio que tuvo niveles medianos del SII de

48.8 (8.6-85.7) y del CIX de 67.0 (45.0-85.8). Las intervenciones principalmente prestadas en unidades sanitarias: parto institucional (SII mediano 46.7 [23.1-63.3] y CIX 11.4 [4.5-23.4]) y cuidados prenatales (SII mediano 26.7 [17.0-47.2] e CIX 10.0 [4.2-17.1]) también tuvieron patrones pro-ricos. En comparación, las intervenciones principalmente prestadas en la comunidad, incluyendo aquellas contra la malaria, fueron distribuidas de modo más equitativo – p. ej. sales de hidratación oral (SII mediano 9.4 [2.9-19.0] y CIX 3.4 [1.3-25.0]) e inmunización contra polio (SII 12.1 [2.3-25.0] y CIX 3.1 [0.5-7.1]). Las diferencias entre los cuatro tipos de canales de prestación en términos de ambos índices de desigualdad fueron significativas (SII p=0.0052; CIX p=0.0048).

**Interpretación.** Las intervenciones prestadas a menudo en el nivel comunitario son generalmente distribuidas de una manera más equitativa que aquellas principalmente prestadas en unidades sanitarias, o aquellas que exigen cambios en el entorno del hogar. Los responsables de la formulación de políticas públicas necesitan comprender el papel de los canales de prestación comunitarios para promover un acceso más equitativo a todas las intervenciones de SRMNN.

**Financiamento** Bill and Melinda Gates Foundation y Wellcome Trust

**Copyright** © 2021 El(los) Autor(es). Publicado por Elsevier Ltd. Este es un articulo de Acceso Abierto bajo la licencia CC BY 4.0.
