# Supplementary material for: Delivery channels and socioeconomic inequalities in coverage of reproductive, maternal, newborn, and child health interventions: analysis of 36 cross-sectional surveys in low-income and middle-income countries
Source: Lancet Glob Health. 2021 May 26;9(8):e1101–9. doi: 10.1016/S2214-109X(21)00204-7 (PMC8295042; doi:10.1016/S2214-109X(21)00204-7)
Supplement: Supplementary appendix 4 [file mmc4.pdf]

THE LANCET
Global Health

Supplementary appendix 4

This appendix formed part of the original submission and has been peer reviewed. We post it as supplied by the authors.

Supplement to: Leventhal DGP, Crochemore-Silva I, Vidaletti LP, Armenta-Paulino N, Barros AJD, Victora CG. Delivery channels and socioeconomic inequalities in coverage of reproductive, maternal, newborn, and child health interventions: analysis of 36 cross-sectional surveys in low-income and middle-income countries. *Lancet Glob Health* 2021; published online May 26. http://dx.doi.org/10.1016/S2214-109X(21)00204-7.

Supplementary Materials

Delivery channels and the magnitude of socioeconomic inequalities in coverage of RMNCH interventions: Analysis of 36 cross-sectional surveys in low- and middle-income countries

Contents

Supplementary Figure 1. World map showing countries included in analyses (n=36). 2

Supplementary Figure 2. Coverage (medians and interquartile ranges) with 20 RMNCH interventions in low-income countries, 2010 or later. 3

Supplementary Figure 3. Coverage (medians and interquartile ranges) with 20 RMNCH interventions, 2010 or later, 21 countries with endemic malaria. 4

Supplementary Figure 4. Socioeconomic inequalities in coverage of 20 RMNCH interventions in LMICs, 2010 or later, 21 countries with endemic malaria 5

Supplementary Figure 5. Socioeconomic inequalities in coverage of 20 RMNCH interventions in LMICs, 2010 or later, all available data points from 104 countries, most recent survey in each country. 6

Supplementary Table 1. Definitions, target age groups, and position along the continuum of care of RMNCH indicators 7

Supplementary Table 2. Distribution of surveys according to type and date, world regions and World Bank income group, and number of indicators available. 9

Supplementary Table 3. Magnitude of coverage and socioeconomic inequalities in RMNCH indicators. 12

Supplementary Table 4. Differences in coverage, absolute (SII), and relative (CIX) inequality among RMNCH indicators between low-income (LIC) and middle-income countries (MIC). 13

Supplementary Table 5. Magnitude of coverage and socioeconomic inequalities in RMNCH indicators in countries with endemic malaria. 14

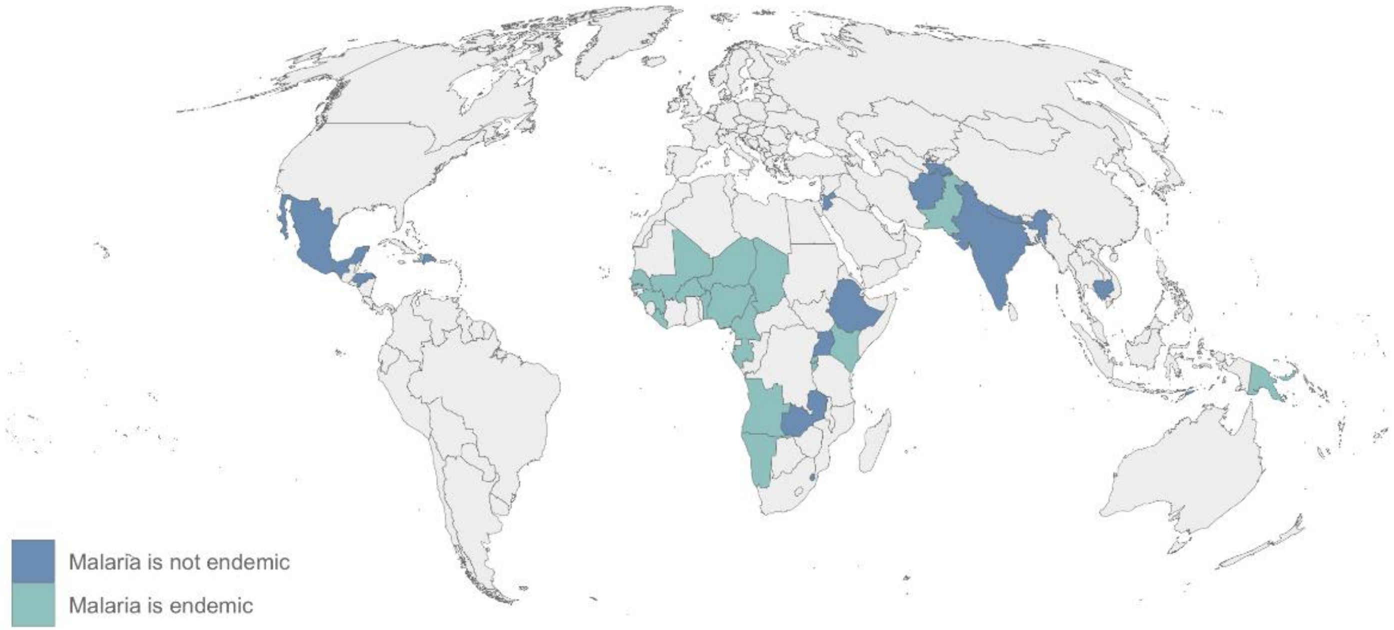

Supplemen/tary Figure 1. World map showing countries included in analyses (n=36).
Countries with endemic malaria appear in turquoise (n=21), while all others appear in blue.

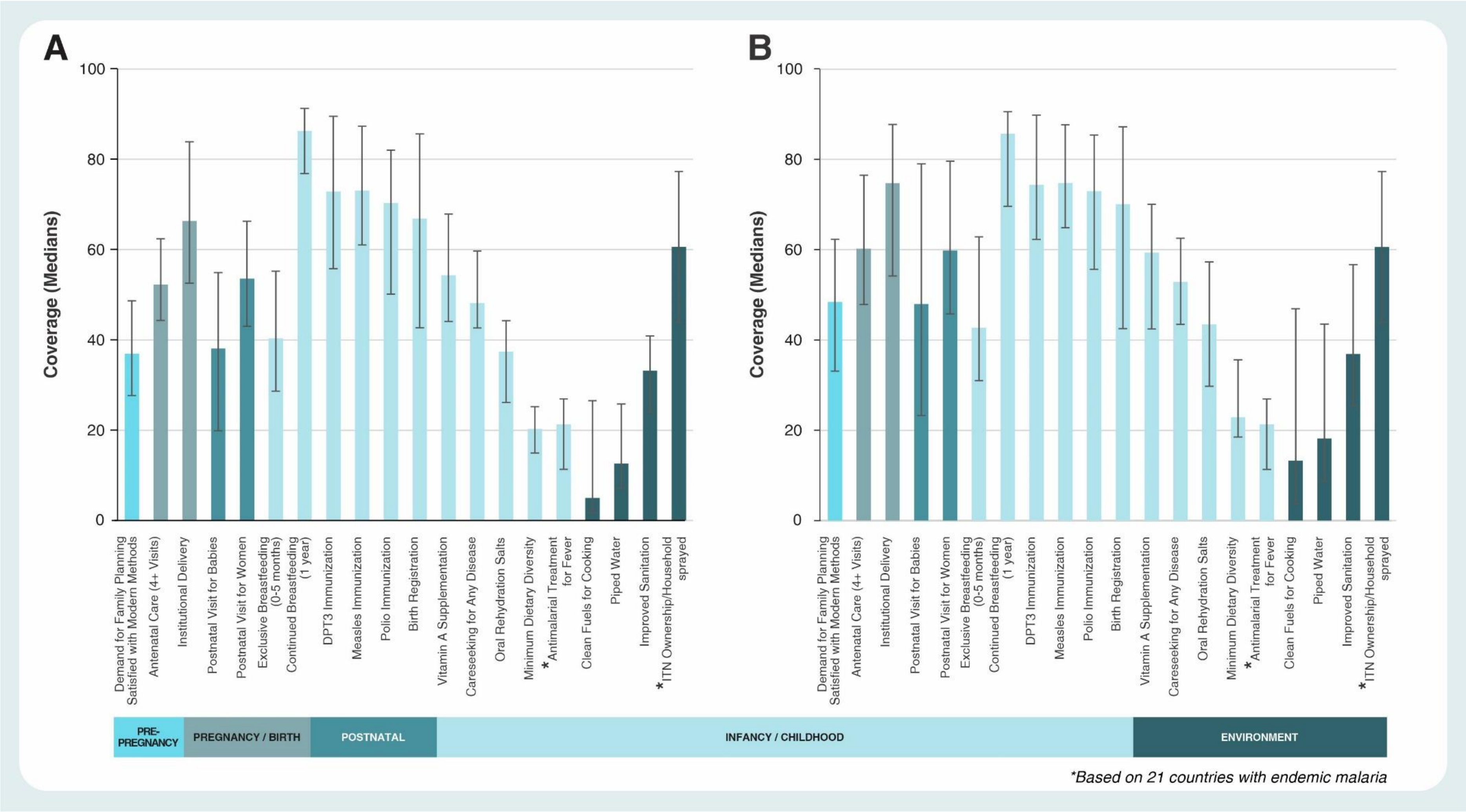

Supplementary Figure 2. Coverage (medians and interquartile ranges) with 20 RMNCH interventions in low-income countries, 2010 or later. A) Low-income countries, n=17 countries. B) Middle income countries, n=19 countries.

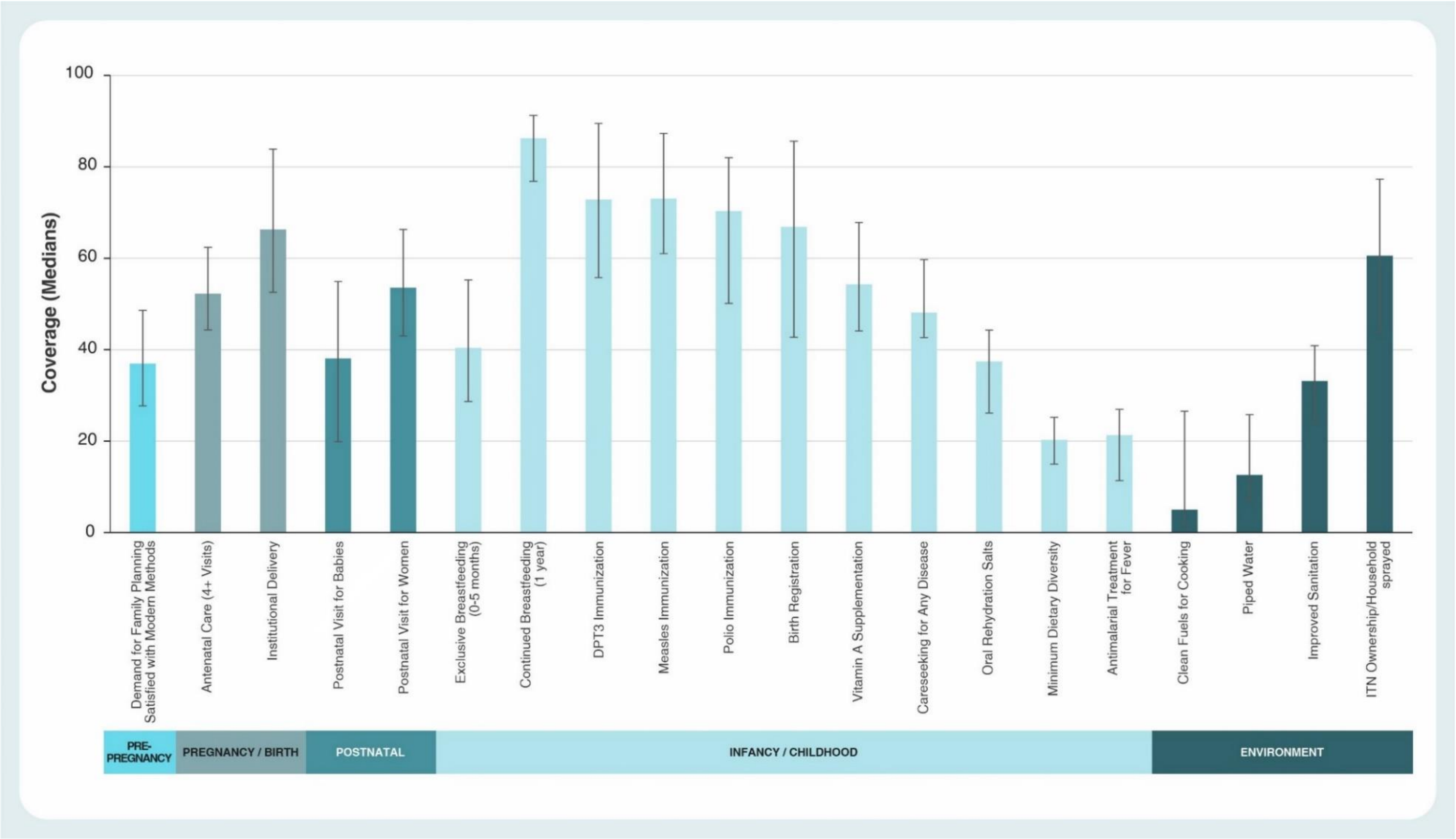

Supplementary Figure 3. Coverage (medians and interquartile ranges) with 20 RMNCH interventions, 2010 or later, 21 countries with endemic malaria.

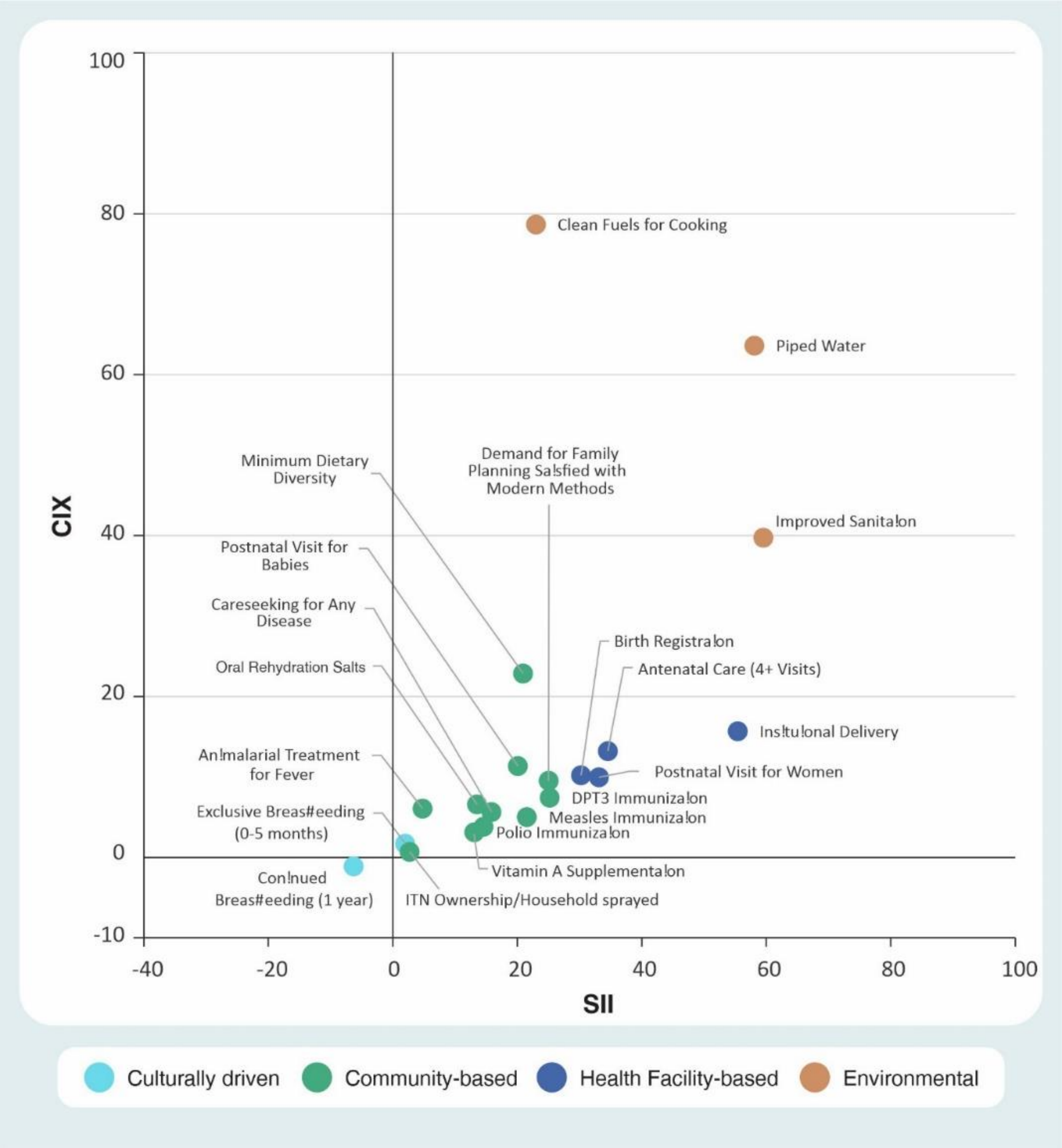

Supplementary Figure 4. Socioeconomic inequalities in coverage of 20 RMNCH interventions in LMICs, 2010 or later, 21 countries with endemic malaria.

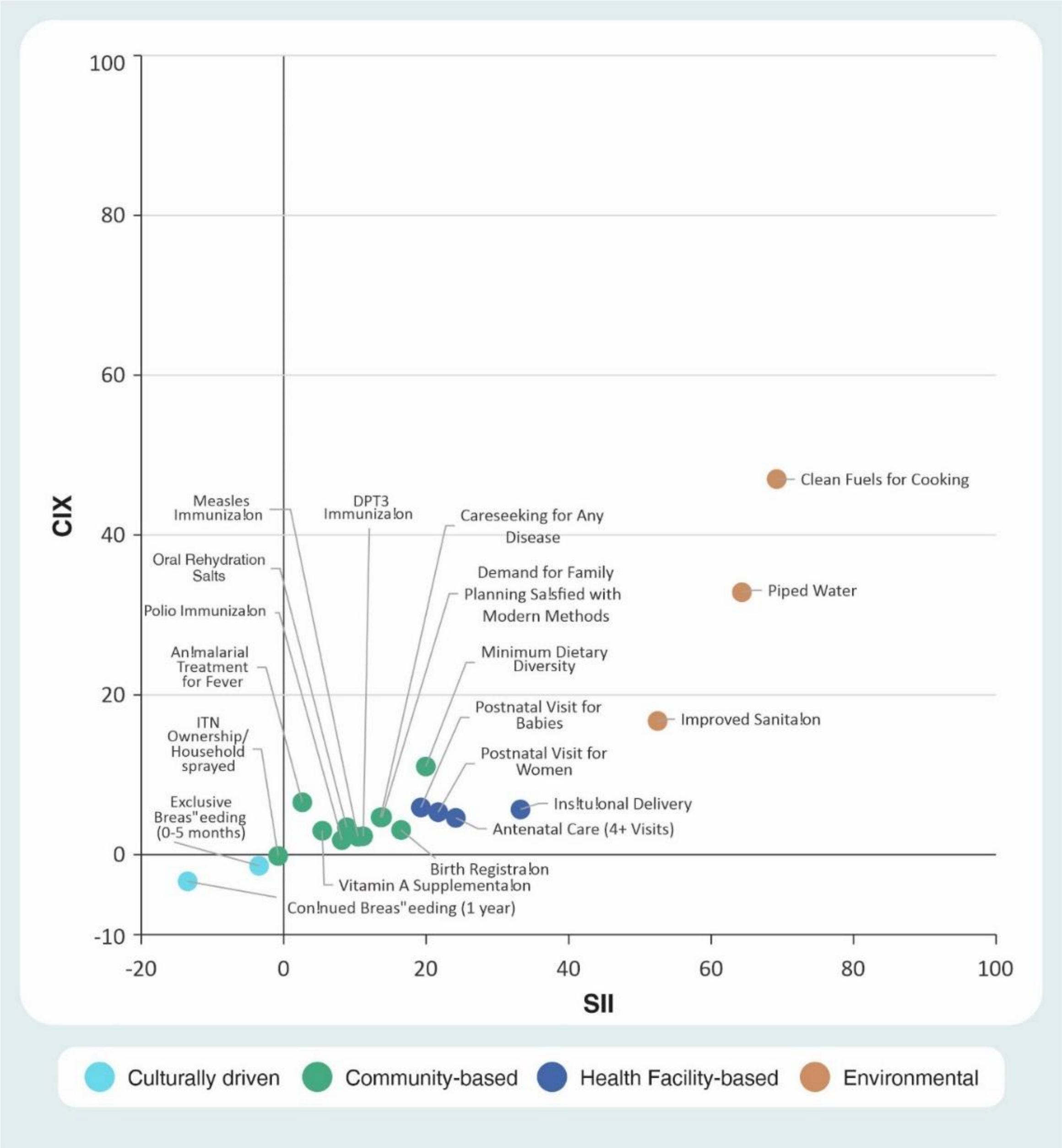

Supplementary Figure 5. Socioeconomic inequalities in coverage of 20 RMNCH interventions in LMICs, 2010 or later, all available data points from 104 countries, most recent survey in each country.

Supplementary Table 1. Definitions, target age groups, and position along the continuum of care of RMNCH indicators

Group	Indicator	Definition	Target Age Group
Environmental	Clean fuels for cooking	Primary reliance on clean fuels for cooking	All
Environmental	Improved sanitation	Proportion of household members with improved sanitation	All
Environmental	Piped water	Proportion of households with piped water connection	All
Health facility-based	Antenatal care (4+ visits)	Proportion of women with at least 4 antenatal care visits by a skilled provider during pregnancy	Women aged 15-49 years
Health facility-based	Institutional delivery	Proportion of children born in a health facility	All live births within the last 2-5 years
Health facility-based	Postnatal visit for women	Proportion of women who received postnatal care within 2 days of delivery	Women aged 15-49
Health facility-based	Birth registration	Proportion of live births that were registered, with or without a birth certificate	All live births within the last 5 years
Community-based	Demand for family planning satisfied with modern methods	Proportion of women married or in union who need contraception and who are using (or whose partner is using) any modern method[1]	Women aged 15-49 years
Community-based	Postnatal visit for babies	Proportion of babies who received postnatal care within 2 days of birth	Children born within the last 2 years
Community-based	DPT3 immunization	Proportion of children who received DPT3 immunization	Children aged 12-23 months, 15-26 months, or 18-29 months, depending on survey reference

[1] Including oral contraceptive pills, injectables, diaphragms, cervical caps, vaginal rings, condoms (male and female), spermicidal agents, patch contraception, emergency contraceptive pills, IUDs, implants and sterilization (male and female). Source: Hubacher D, Trussell J. A definition of modern contraceptive methods. Contraception. 2015 Nov;92(5):420-1.

Community-based	Measles immunization	Proportion of children who received measles immunization	Children aged 12-23 months, 15-26 months, or 18-29 months, depending on survey reference
Community-based	Polio immunization	Proportion of children who received polio immunization	Children aged 12-23 months, 15-26 months, or 18-29 months, depending on survey reference
Community-based	Vitamin A supplementation	Proportion of children aged 6-59 months who received vitamin A supplementation	Children aged 6-59 months
Community-based	Careseeking for any disease	Proportion of children under 5 years who sought healthcare for any disease in the last 2 weeks	Children aged 0-59 months
Community-based	Oral rehydration salts	Proportion of children under 5 years with diarrhea in the last 2 weeks who received oral rehydration salts	Children aged 0-59 months
Community-based	Minimum dietary diversity	Proportion of children 6-23 months of age who received food from at least 5 of 8 food groups during the previous day	Children aged 6-23 months
Community-based	Antimalarial treatment for fever	Proportion of children with fever treated with antimalarial medication	Children aged 0-59 months
Community-based	ITN ownership/household sprayed	Ownership of at least 1 insecticide-treated net (ITN) OR household sprayed	All
Culturally driven	Exclusive breastfeeding (0-5 months)	Proportion of children exclusively breastfed (0-5 months)	Young children aged 0-5 months, living with their mother
Culturally driven	Continued breastfeeding (12-15 months)	Proportion of children aged 12-15 months who continue to breastfeed	Youngest children aged 12-15 months, living with their mother

Supplementary Table 2. Distribution of surveys according to type and date, world regions and World Bank income group, and number of indicators available.

Country	Region	Country income group	Type of survey	Date	Number of indicators*
Afghanistan	South Asia	Low income	DHS	2015	18
Angola	Eastern and Southern Africa	Lower-middle income	DHS	2015	20
Benin	West and Central Africa	Low income	DHS	2017	20
Burkina Faso	West and Central Africa	Low income	DHS	2010	20
Burundi	Eastern and Southern Africa	Low income	DHS	2016	20
Cambodia	East Asia and the Pacific	Lower-middle income	DHS	2014	18
Cameroon	West and Central Africa	Lower-middle income	DHS	2018	20
Chad	West and Central Africa	Low income	DHS	2014	20
Comoros	Eastern and Southern Africa	Low income	DHS	2012	20
Dominican Republic	Latin America and Caribbean	Upper-middle income	MICS	2014	18
Eswatini	Eastern and Southern Africa	Lower-middle income	MICS	2014	18
Ethiopia	Eastern and Southern Africa	Low income	DHS	2016	18
Gabon	West and Central Africa	Upper-middle income	DHS	2012	20
Guinea	West and Central Africa	Low income	DHS	2018	20
Haiti	Latin America and Caribbean	Low income	DHS	2016	20

Honduras	Latin America and Caribbean	Lower-middle income	DHS	2011	18
India	South Asia	Lower-middle income	DHS	2015	18
Jordan	Middle East and North Africa	Upper-middle income	DHS	2017	18
Kenya	Eastern and Southern Africa	Lower-middle income	DHS	2014	20
Liberia	West and Central Africa	Low income	DHS	2013	20
Maldives	South Asia	Upper-middle income	DHS	2016	18
Mali	West and Central Africa	Low income	DHS	2018	20
Mexico	Latin America and Caribbean	Upper-middle income	MICS	2015	18
Namibia	Eastern and Southern Africa	Upper-middle income	DHS	2013	20
Nepal	South Asia	Low income	DHS	2016	18
Niger	West and Central Africa	Low income	DHS	2012	20
Nigeria	West and Central Africa	Lower-middle income	DHS	2018	20
Pakistan	South Asia	Lower-middle income	DHS	2017	20
Papua New Guinea	East Asia and the Pacific	Lower-middle income	DHS	2016	20
Rwanda	Eastern and Southern Africa	Low income	DHS	2014	20
Sao Tome and Principe	West and Central Africa	Lower-middle income	MICS	2014	20
Senegal	West and Central Africa	Low income	DHS	2017	20
Tajikstan	Eastern Europe and Central Asia	Low income	DHS	2017	18

Timor-Leste	East Asia and the Pacific	Lower-middle income	DHS	2016	18
Uganda	Eastern and Southern Africa	Low income	DHS	2016	18
Zambia	Eastern and Southern Africa	Lower-middle income	DHS	2018	18

(*) Countries with endemic malaria had information on 20 indicators, and the remaining countries on 18 indicators.

Supplementary Table 3. Magnitude of coverage and socioeconomic inequalities in RMNCH indicators.
IQR = Interquartile Range, SD = Standard Deviation. *Data available from 21 countries with endemic malaria.

Indicators	N	Coverage (%)					Slope index of inequality (SII)					Concentration index (CIX)				
		median	IQR	mean	SD	95% IC	median	IQR	mean	SD	95% IC	median	IQR	mean	SD	95% IC
Clean fuels for cooking	36	13.3	3.8;46.9	29.0	31.9	18.2;39.7	48.8	8.6;85.7	48.4	37.4	35.7;61.0	67.0	45.0;85.8	59.5	30.3	49.2;69.7
Improved sanitation	36	36.9	25.4;56.7	44.1	26.4	35.2;53.1	57.7	41.0;75.2	54.4	26.4	45.5;63.3	33.5	16.9;44.7	33.1	21.0	26.0;40.2
Piped water	36	18.2	8.8;43.5	29.1	26.1	20.3;37.9	57.3	35.1;73.6	53.5	26.2	44.6;62.3	58.5	27.6;73.2	50.6	27.6	41.3;60.0
Antenatal care (4+ visits)	36	60.2	47.9;76.5	60.1	19.5	53.5;66.7	26.7	17.0;47.2	31.2	19.7	24.5;37.8	10.0	4.2;17.1	10.9	8.0	8.1;13.6
Institutional delivery	36	74.8	54.2;87.7	69.3	21.7	62.0;76.7	46.7	23.1;63.3	43.7	24.0	35.6;51.9	11.4	4.5;23.4	14.7	11.6	10.8;18.6
Postnatal visit for women	36	59.8	45.8;79.6	60.5	22.1	53.0;68.0	29.8	13.7;44.3	29.6	17.2	23.8;35.5	8.0	3.6;16.5	11.3	9.4	8.2;14.5
Birth registration	36	70.1	42.5;87.2	64.2	28.3	54.6;73.8	28.4	10.2;40.0	27.2	20.3	20.3;34.1	7.8	2.6;15.9	12.0	12.8	7.6;16.3
Demand for family planning satisfied	36	48.4	33.1;62.3	48.7	19.6	42.0;55.3	15.2	8.5;28.0	18.6	15.5	13.3;23.8	6.0	2.7;11.7	8.9	9.6	5.7;12.2
Postnatal visit for babies	36	48.0	23.3;79.0	49.7	28.6	40.0;59.4	17.8	8.2;37.0	24.9	19.1	18.5;31.4	9.5	4.1;16.2	11.6	10.0	8.3;15.0
DPT3 immunization	36	74.4	62.2;89.8	74.1	17.9	68.1;80.2	17.6	4.3;33.0	19.8	18.7	13.4;26.1	4.2	0.7;8.4	5.9	6.5	3.7;8.1
Measles immunization	36	74.8	64.9;87.7	75.0	14.1	70.3;79.8	16.5	1.1;31.0	18.2	19.1	11.7;24.6	4.0	0.3;8.0	5.1	5.8	3.1;7.0
Polio immunization	36	72.9	55.6;85.4	71.4	16.8	65.7;77.1	12.1	2.3;25.0	13.4	16.4	7.9;18.9	3.1	0.5;7.1	4.0	5.6	2.1;5.9
Vitamin A supplementation	36	59.4	42.5;70.1	52.7	22.7	45.0;60.4	5.5	-0.7;16.2	8.0	12.9	3.6;12.4	3.6	-0.1;5.7	3.6	7.1	1.2;6.0
Careseeking for any disease	36	52.9	43.5;62.5	53.0	13.5	48.5;57.6	12.1	3.3;23.9	12.2	13.4	7.6;16.7	4.7	0.9;8.5	4.9	5.3	3.1;6.6
Oral rehydration salts	36	43.4	29.8;57.3	44.4	17.1	38.6;50.1	9.4	2.9;19.0	9.8	13.2	5.4;14.3	3.4	1.3;8.5	4.9	6.2	2.8;7.1
Minimum dietary diversity	36	22.9	18.5;35.6	27.8	15.5	22.5;33.0	20.4	10.4;28.9	21.5	12.1	17.4;25.6	15.7	8.8;24.6	17.0	10.6	13.4;20.6
Antimalarial treatment for fever*	21	21.3	11.4;27.0	22.7	14.6	16.0;29.3	4.8	-4.7;15.4	4.7	12.5	-1.0;10.4	6.1	-7.8;10.0	0.5	17.6	-7.5;8.5
ITN ownership /household sprayed*	21	60.6	43.9;77.3	59.8	23.1	49.2;70.3	2.7	-16.7;16.3	1.8	22.1	-8.3;11.9	0.7	-5.4;5.8	0.3	8.1	-3.4;4.0
Exclusive breastfeeding (0-5 months)	36	42.7	31.0;62.9	44.9	21.5	37.7;52.2	-0.8	-15.3;4.3	-4.0	15.2	-9.1;1.1	-0.3	-5.8;3.0	-1.4	7.8	-4.0;1.3
Continued breastfeeding (1 year)	36	85.7	69.6;90.6	77.6	17.8	71.6;83.6	-11.1	-34.1;-2.4	-17.3	17.7	-23.3;-11.3	-2.1	-8.3;-0.7	-4.7	5.3	-6.5;-2.9

Supplementary Table 4. Differences in coverage, absolute (SII), and relative (CIX) inequality among RMNCH indicators between low-income (LIC) and middle-income countries (MIC).

Indicators	Low-income countries (LIC)			Middle-income countries (MIC)			Differences (LIC-MIC)		
	Coverage	SII	CIX	Coverage	SII	CIX	Coverage	SII	CIX
Clean fuels for cooking	4.5	21.4	85.6	43.9	79.9	49.3	-39.3	-58.5	36.2
Improved sanitation	27.2	54.1	39.8	48.1	59.4	31.1	-20.9	-5.3	8.7
Piped water	9.3	50.0	73.3	30.1	61.9	41.5	-20.9	-11.9	31.9
Antenatal care (4+ visits)	49.2	26.4	13.0	75.7	34.0	7.8	-26.5	-7.6	5.1
Institutional delivery	66.3	50.8	14.6	83.2	37.0	7.0	-16.9	13.8	7.5
Postnatal visit for women	51.1	28.1	9.6	69.0	33.8	7.9	-17.9	-5.6	1.8
Birth registration	63.9	28.3	8.6	73.3	32.3	7.7	-9.4	-4.0	0.9
Demand for family planning satisfied with modern methods	39.4	18.9	9.2	55.0	13.5	3.3	-15.6	5.4	5.9
Postnatal visit for babies	42.9	19.4	9.9	64.2	15.7	7.0	-21.2	3.7	2.9
DPT3 immunization	73.1	22.0	6.4	78.7	14.4	3.9	-5.7	7.7	2.6
Measles immunization	74.2	16.9	4.4	78.6	12.9	3.4	-4.4	4.0	1.0
Polio immunization	70.3	11.2	3.1	74.6	13.1	2.5	-4.2	-1.9	0.6
Vitamin A supplementation	60.2	13.3	3.7	54.3	4.6	3.6	5.9	8.6	0.1
Careseeking for any disease	46.5	15.3	5.7	61.2	5.2	1.7	-14.7	10.1	3.9
Oral rehydration salts	37.0	8.6	3.5	49.1	12.0	2.8	-12.2	-3.3	0.8
Minimum dietary diversity	20.3	18.2	22.9	32.2	24.6	11.2	-11.9	-6.4	11.7
Antimalarial treatment for fever*	22.0	5.9	6.7	21.3	2.9	2.4	0.7	3.0	4.3
ITN ownership/household sprayed*	61.0	11.5	2.7	59.1	-0.4	0.4	1.9	11.8	2.3
Exclusive breastfeeding (0-5 months)	41.5	0.0	0.4	48.5	-1.9	-1.9	-6.9	1.9	2.3
Continued breastfeeding (1 year)	90.8	-5.7	-1.1	69.6	-32.5	-7.8	21.2	26.8	6.7

Supplementary Table 5. Magnitude of coverage and socioeconomic inequalities in RMNCH indicators in countries with endemic malaria.
IQR = Interquartile Range, SD = Standard Deviation.

Indicators	N	Coverage (%)					Slope index of inequality (SII)					Concentration index (CIX)				
		median	IQR	mean	SD	95% CI	Median	IQR	mean	SD	95% CI	median	IQR	mean	SD	95% CI
Clean fuels for cooking	21	5.0	1.8;26.5	16.3	22.0	6.3;26.3	23.0	8.1;80.3	41.2	37.5	24.1;58.2	78.7	51.8;88.2	69.0	26.1	57.1;80.8
Improved sanitation	21	33.2	24.0;40.9	32.8	16.0	25.5;40.1	59.6	54.1;79.1	64.2	17.0	56.5;72.0	39.8	28.5;55.1	42.3	18.4	33.9;50.7
Piped water	21	12.6	7.2;25.8	20.9	21.4	11.1;30.6	58.1	38.2;66.7	51.7	25.1	40.3;63.1	63.6	30.0;80.6	58.2	26.3	46.2;70.2
Antenatal care (4+ visits)	21	52.2	44.3;62.4	53.6	14.7	47.0;60.3	34.6	25.2;48.4	37.1	19.5	28.2;45.9	13.2	7.8;17.3	12.9	7.1	9.6;16.1
Institutional delivery	21	66.3	52.6;83.9	64.3	20.8	54.9;73.7	55.4	35.4;69.1	51.1	20.7	41.7;60.6	15.7	8.6;24.6	17.6	11.2	12.5;22.7
Postnatal visit for women	21	53.6	43.0;66.3	53.7	18.0	45.5;61.9	33.1	16.1;48.7	33.6	17.0	25.8;41.3	9.9	6.2;17.1	13.1	8.9	9.0;17.1
Birth registration	21	66.9	42.7;85.6	63.2	26.4	51.2;75.2	30.3	18.1;44.8	33.8	20.4	24.5;43.0	10.2	5.1;20.0	13.3	11.7	8.0;18.6
Demand for family planning satisfied with modern methods	21	37.0	27.7;48.6	39.9	16.3	32.5;47.3	25.1	12.0;32.4	23.9	15.9	16.6;31.1	9.5	5.8;16.7	12.9	10.6	8.1;17.7
Postnatal visit for babies	21	38.1	19.9;54.9	39.4	23.0	28.9;49.8	20.1	11.9;52.4	29.2	21.2	19.6;38.9	11.3	7.8;18.4	13.6	8.5	9.7;17.4
DPT3 immunization	21	72.9	55.8;89.5	70.9	20.1	61.7;80.0	25.2	11.6;38.4	25.7	19.0	17.0;34.3	7.4	2.2;10.2	7.8	7.1	4.6;11.1
Measles immunization	21	73.1	61.0;87.3	72.8	15.4	65.8;79.8	21.6	11.3;35.3	24.3	19.8	15.2;33.3	5.0	3.2;10.7	6.9	6.4	4.0;9.8
Polio immunization	21	70.3	50.1;82.0	67.6	18.8	59.0;76.2	13.1	4.4;28.6	15.7	17.9	7.5;23.9	3.1	1.0;7.6	5.0	6.6	2.0;7.9
Vitamin A supplementation	21	54.3	44.1;67.8	53.0	21.4	43.2;62.7	14.6	2.4;22.0	11.7	14.2	5.2;18.1	3.8	0.8;6.3	5.7	7.9	2.1;9.3
Careseeking for any disease	21	48.1	42.7;59.7	48.2	12.6	42.5;54.0	15.9	10.1;24.5	17.1	11.4	11.9;22.3	5.6	4.2;10.6	6.8	5.0	4.5;9.1
Oral rehydration salts	21	37.4	26.1;44.3	37.2	14.6	30.5;43.9	13.5	3.7;20.7	12.7	11.7	7.4;18.0	6.6	2.2;11.2	7.1	6.6	4.1;10.1
Minimum dietary diversity	21	20.3	15.0;25.2	20.7	8.9	16.7;24.8	20.9	17.3;28.0	23.2	13.1	17.2;29.2	22.9	11.2;28.0	21.3	10.4	16.6;26.0
Antimalarial treatment for fever*	21	21.3	11.4;27.0	22.7	14.6	16.0;29.3	4.8	-4.7;15.4	4.7	12.5	-1.0;10.4	6.1	-7.8;10.0	0.5	17.6	-7.5;8.5
ITN ownership /household sprayed*	21	60.6	43.9;77.3	59.8	23.1	49.2;70.3	2.7	-16.7;16.3	1.8	22.1	-8.3;11.9	0.7	-5.4;5.8	0.3	8.1	-3.4;4.0
Exclusive breastfeeding (0-5 months)	21	40.4	28.7;55.2	42.3	23.1	31.8;52.8	2.0	-2.4;11.0	0.7	14.3	-5.8;7.2	1.7	-1.0;5.0	1.5	7.1	-1.8;4.7
Continued breastfeeding (1 year)	21	86.3	76.9;91.2	82.8	13.2	76.8;88.8	-6.3	-31.0;-1.6	-15.0	17.8	-23.1;-6.9	-1.1	-7.4;-0.6	-3.8	5.0	-6.1;-1.5
